# An ultrasound visual servoing dual-arm robotics system for needle placement in brachytherapy treatment

**DOI:** 10.3389/frobt.2025.1558182

**Published:** 2025-03-28

**Authors:** Yanlei Li, Zhenyu Lu, Antonia Tzemanaki, Amit Bahl, Raj Persad, Chris Melhuish, Chenguang Yang

**Affiliations:** ^1^ Bristol Robotics Laboratory, Bristol, United Kingdom; ^2^ Department of Engineering Design and Mathematics, Faculty of Environment and Technology, University of the West of England, Bristol, England, United Kingdom; ^3^ School of Automation Science and Engineering, South China University of Technology, Guangzhou, China; ^4^ School of Engineering Mathematics and Technology, University of Bristol, Bristol, England, United Kingdom; ^5^ University Hospitals Bristol NHS Foundation Trust, Bristol, England, United Kingdom; ^6^ School of Electrical Engineering, Electronics and Computer Science, Faculty of Science and Engineering, University of Liverpool, Liverpool, North West England, United Kingdom

**Keywords:** medical robot, prostate brachytherapy, dual arm robotics, ultrasound tracking, camera tracking, fuzzy control, needle steering, visual servo system

## Abstract

The accurate placement of radioactive seeds in prostate brachytherapy is critical to the efficacy of the procedure. Current manual needle insertion methods face challenges, including reduced accuracy due to hand tremors, high dependence on surgeon expertise, and strain during lengthy procedures. Additionally, manual approaches often struggle to adapt to tissue heterogeneities, leading to unsatisfied outcomes. Autonomous needle placement is difficult due to varying tissue parameters. This paper presents an innovative dual-arm visual-servo robotics system for needle steering precision during prostate brachytherapy. The system employs two Franka Emika arms: one for needle insertion and the other for positioning the ultrasound probe. Based on the real-time position feedback, a fuzzy logic controller guides needle steering, and a camera system offers supplementary tracking and safety monitoring. In order to identify the needle tip’s position within tissue, a novel image recognition method which is intuitive to the surgeon is proposed with the use of the ultrasound probe. It is in conjunction with the scanning and control mode of the dual-arm robotic arm to locate the position of the needle tip inside the tissue. The camera system is also unified in the same dual-arm robotic arm coordinate system to monitor the entire needle steering process. By addressing the limitations of manual techniques, including accuracy, efficiency, and adaptability to tissue variations, the proposed system reduces the skill barrier, workload, and potential trauma associated with brachytherapy procedures. Experimental validation on a phantom shows a final needle placement accuracy of 0.285 cm, demonstrating the system’s potential to improve treatment outcomes through precise needle control.

## 1 Introduction

Prostate cancer is one of the most prevalent cancers affecting individuals assigned male at birth, particularly those over the age of 50. In 2017 alone, over 1.2 million new cases of prostate cancer were diagnosed globally (Moreno et al., 2017). Prostate brachytherapy is considered an effective therapeutic option for prostate cancer due to its high success rate (Keyes et al., 2013). The procedure involves the precise delivery of radiation directly to the tumor through the implantation of radioactive seeds, which are placed in or near the tumor using multiple long, flexible, and hollow needles. Initially, the surgeon uses ultrasound imaging to guide the needle into the prostate, visualizing the needle’s target position in real-time. The process begins with the surgeon positioning the ultrasound probe in the perineum to get a clear view of the prostate. After determining the final target position, the needle is carefully inserted through the skin, aiming toward the target tissue. During this process, the surgeon uses a brachytherapy grid to limit any deviation of the needle, ensuring it follows as straight a path as possible toward the target. Despite these precautions, in some cases, deviations remain unforeseen and impossible to properly control even with these measures. Although prostate brachytherapy provides notable clinical benefits, there is still potential for improvement, especially in enhancing the precision of needle placement to optimize radiation distribution according to radiotherapy principles. In recent years, robotic needle steering has emerged as a promising approach to enhance the accuracy and efficacy of minimally invasive procedures such as brachytherapy and biopsy. These interventions rely heavily on precise needle placement to ensure effective treatment or accurate diagnosis. However, traditional manual needle insertion techniques often face challenges due to tissue deformation, needle deflection, and anatomical obstacles (Moreno et al., 2017). To address these issues, researchers have developed various robotic systems that offer improved maneuverability, accuracy, and integration with medical imaging technologies.

Robotic assistance systems have several advantages over traditional manual needle insertion. Guiu et al. (2021) demonstrated that the use of a robotic system can completely eliminate radiation exposure for operators. Additionally, a broad range of orbital and cranio-caudal angulations can theoretically be achieved by robotic devices, allowing for intricate angle insertions. Importantly, these systems can minimize the impact of operator experience, as shown by the experiments by Guiu et al. where no significant differences in insertion accuracy were found between novice and experienced physicians using the robotic system (Guiu et al., 2021).

Baker et al. (1999) introduced electronic beam steering technology in ultrasound-guided breast interventions, which improved needle visibility by increasing the beam’s angle of incidence from 66° to 84°. Phee et al. (2005) developed a robotic system for transperineal prostate biopsy that combined 3D ultrasound imaging with automated needle guidance, enabling more precise needle placement and multiple biopsies through a single entry point. In 2009, a five-degree-of-freedom (5-DOF) micromanipulator had been developed by Bassan et al. (2009) to assist surgeons in prostate brachytherapy by enabling automated insertion and rotation of the needle base. Long et al. (2012) developed the PROSPER robot, featuring seven degrees of freedom and integrating intra-operative prostate tracking to improve placement accuracy. The system achieved an average final positioning error of 2.73 mm in prostate biopsy and brachytherapy scenarios. Despite this improvement, the system relies on rigid tracking mechanisms, which may struggle with tissue heterogeneity. Burrows et al. (2013) developed a biologically inspired multi-segment needle capable of 3D steering, achieving significant reductions in the mean radius of curvature compared to standard needles. Khadem et al. (2017) introduced notched steerable needles, optimized using finite element modeling, which demonstrate enhanced deflection capabilities and the ability to navigate around structures like the pubic arch. Similarly, Qi et al. (2021) presented a novel telescopic steerable robotic needle, combining a flexible inner tube with a rigid outer tube to achieve follow-the-leader motion in soft tissue. However, the applicability of the above mechanism in clinical settings remains limited due to its complex actuation and control requirements. Halima et al. (2021) developed a 6-DOF parallel co-manipulated robot with integrated gravity compensation, offering a large singularity-free workspace. Pei et al. (2019) proposed a miniature robotic needling device incorporating force feedback and predictive control based on mechanical models of needle behavior to minimize deflection. Bernardes et al. (2023) developed a data-driven adaptive needle insertion assist method for transperineal prostate interventions, offering a flexible approach that can be adapted to varying tissue properties. Koskinopoulou et al. (2023) developed a dual-arm collaborative robotic system for autonomous central venous access, integrating ultrasound imaging and electrical bioimpedance (EBI) sensing. One robot arm controls the ultrasound probe for vessel localization, while the other guides needle insertion with real-time EBI feedback. The experiment demonstrated an RMS positioning error of 1.71 mm during phantom trials, highlighting its potential for precise, autonomous medical interventions. Systems like Pei et al. (2019), Bernardes et al. (2023) and Koskinopoulou et al. (2023) focus on force feedback and adaptive methods but require further refinement to address tissue deformation and deflection effectively.

Various imaging modalities support medical navigation procedures. Ultrasound guidance shows extensive applications in biopsy operations (Chen et al., 2017). Anatomical dose planning is achievable through CT and MRI technologies. These imaging methods enhance procedural efficiency. The cost-effective nature of ultrasound makes it the preferred choice for biopsies. Recent studies have extensively examined all three imaging approaches. For instance, Abdullah et al. (2015) developed a robotic system for CT-guided thermal ablation of liver tumors, achieving a mean accuracy of 2.5 mm. Hiraki et al. (2018) designed a robotic system for inserting various types of ablation needles under CT guidance, with a mean accuracy of 2.8 mm. The CT-guided robotic system proposed by Ben-David et al. (2018) demonstrated high accuracy in animal experiments, with a mean error of 2.3 mm. However, Compared to ultrasound, CT exposes patients to ionizing radiation which makes it less suitable for repeated use. And MRI systems are costly and require specialized equipment, limiting their clinical applicability in resource-constrained settings. Ultrasound (US) imaging, on the other hand, provides a radiation-free and cost-effective alternative. However, using ultrasound for visual servoing in clinical settings is challenging due to certain factors. Ultrasound images can be affected by factors like physical tissue parameters, probe positioning, and acoustic interference which makes it difficult to accurately track the needle tip in real-time. This makes it challenging to use effectively in clinical practice.

These systems have shown promising results in improving insertion accuracy and reducing the number of needle adjustments compared to manual insertions (Cornelis et al., 2015). A needle guidance system was proposed by Abayazid et al. (2014). Surgeons could receive tactile and visual feedback from this procedure that combines ultrasound with vibration-based imaging feedback. The device kept the needle rotating at a specific pace during the procedure, enabling the surgeon to concentrate only on manipulating the needle’s rotation, improving their capacity to evaluate conditions within the tissue. A framework for predicting system outputs using the minimal potential energy method was proposed by Khadem et al. (2016). This method addressed the problem of estimating diseased states in the absence of precise knowledge of organ characteristics by integrating force sensor data from needle insertions with ultrasonic probe imaging information to estimate local tissue attributes. However, these methods still rely heavily on manual operation, requiring a high level of surgeon expertise. And they could still face challenges when dealing with tissue deformation and heterogeneity, which are common in clinical settings. Furthermore, Šuligoj et al. (2022) integrated machine vision techniques with ultrasound imaging technologies to let micro-robots detect and follow particles inside blood arteries. Rabiei and Konh (2022) designed a portable robotic system incorporating a tendon-driven active needle with automated ultrasound tracking. de Vries et al. (2023) presented an MR-compatible robotic system for MRI-guided HDR prostate brachytherapy, enabling accurate needle placement within the MRI environment. Other advancements in robotic systems for prostate interventions include the development of needle release mechanisms enabling multiple insertions (Chen et al., 2017), CBCT-guided robotic assistance (Pfeil et al., 2019), and systems for fully automated brachytherapy seed placement (Zhu et al., 2019). These advancements collectively aim to improve the accuracy of needle placement, enhance the ability to reach difficult targets, and increase the overall efficacy of brachytherapy and biopsy procedures. However, the aforementioned systems are not fully automated needle steering systems. Although they reduce the surgeon’s workload during surgery, they still require the surgeon to manually insert the needle or operate a teleoperation controller to steer the needle within the tissue.

In summary, while current imaging modalities and robotic systems have made significant improvement in needle placement accuracy, they still face challenges such as radiation risks associated with CT and the high cost of MRI systems. Besides, existing robotic systems often lack robust adaptability to tissue deformation and variations in clinical environments. Additionally, most of the robotics systems are not fully automated and still require surgeon’s operation during the surgery. To address these limitations, this paper proposes a dual-arm robotics system with ultrasound and camera to automatic steer the needle in brachytherapy in prostate cancer treatment. The key contributions of this paper are the development of a novel automated dual-arm visual-servo surgical robotics system and its three integrated subsystems: a multi-modal visual tracking system, a specially designed control system, and a coordinated dual-arm robotic framework. The visual tracking system combines an intuitive image recognition method using an ultrasound probe with a camera-based tracking system. The ultrasound probe, in conjunction with the scanning and control mode of the dual-arm robotic arm, accurately locates the needle tip inside the tissue, while the camera system provides complementary safety monitoring throughout the steering process. To address the challenge of unknown tissue parameters, a custom-made fuzzy logic control algorithm is implemented for adaptive and precise needle steering. Together, these components enhance the system’s reliability, safety, and accuracy, making it a significant advancement in brachytherapy procedures. The structure of the paper is as follows. Section 2 introduces the overall system design and Section 3 provides a detailed description of its three subsystems. Section 4 outlines the experimental design and evaluates the results obtained from the experiments. Section 5 includes the conclusions and suggestions for future work.

## 2 Overall system design

The system is composed of three primary subsystems: a visual tracking system, a robotic control system, and a portable ultrasound scanning probe. These subsystems work in coordination to provide precise guidance for the brachytherapy needle during the insertion process while offering continuous visual feedback to the clinician via a screen. Such feedback is essential for real-time tracking of the trajectory of the needle’s tip both inside and outside the experimental tissue as well as visual monitoring and assurance to the clinician. The system’s control and steering capabilities are enabled by two Franka Emika robotic arms. One arm is fitted with a custom-designed end-effector specifically designed to stabilize and steer the brachytherapy needle. The second robotic arm is similarly equipped with a custom-made end-effector designed to secure the portable ultrasound device, thereby enabling real-time tracking of the needle tip’s position within the phantom during the insertion procedure. This configuration ensures effective coordination between needle guidance and imaging, facilitating accurate targeting and minimizing procedural errors.

**FIGURE 1:**
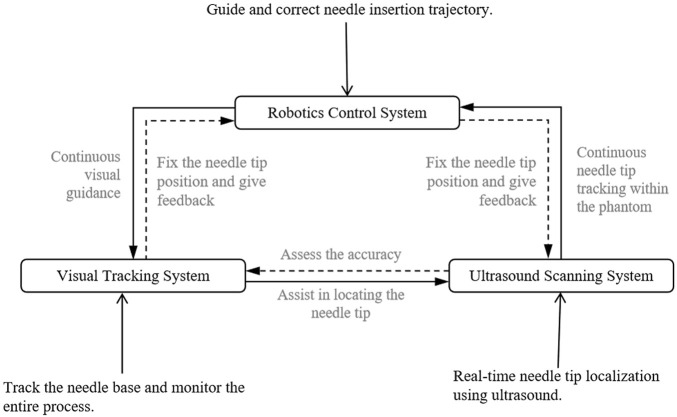
Overall system design and the interrelationships between subsystems.

The workflow is shown in Figure 1, explaining how the system tracks and regulates the insertion of needles during the process. Before beginning surgery, the surgeon needs to specify a 3D target point which should be inside the tissue. This point is formed of a 2D coordinate and a depth that the needle tip needs to reach according to the patient’s treatment plan. The system automatically chooses the best insertion point to begin the needle’s insertion once the goal point has been established. It then creates the optimal needle tip trajectory from the starting point of insertion to the target. The system then uses this predefined trajectory as a guide to monitor and control the needle’s tip, making sure that the needle tip stays precisely on the intended path for the duration of the insertion procedure which to guarantee the accurate positioning and placement of the needle tip. The two robotic arms are controlled by the system asynchronously. Each arm is moving forward 1 cm at a time during the insertion procedure. The movement begins with the robotic arm holding the ultrasound instrument, which is then followed by the robotic arm manipulating the needle. As will be explained in more detail in the next section, this sequence enables the ultrasound to catch the needle tip position effectively. The ultrasound system sends the control system the real-time coordinates as soon as it locates the needle tip precisely. Then, in order to assess any deviations, the control system compares the needle’s present position with the corresponding point on the specified trajectory. The control system uses this analysis to assess if the observed deviation may be corrected by adjusting the position of the needle base in order to compensate the bias between the needle tip and the intended path. Subsequently, a new command is sent to the low-level control system to adjust the needle’s orientation after the next 1-cm movement of the ultrasound-holding robotic arm. Throughout the insertion procedure, the camera keeps track of the needle base, which is attached to the robotic arm’s end effector. Furthermore, the surgeon can use the camera system to monitor the patient’s status during surgery, ensuring complete visual supervision of the procedure.

## 3 Subsystems and methodology

### 3.1 The ultrasound tracking system

The needle insertion operation will unavoidably cause structural changes to the phantom’s tissue during the whole procedure. To overcome this issue, this subsystem employs a mathematical morphology-inspired needle localization method in conjunction with conventional image processing techniques. The main idea behind this method is to transfer the task of needle localization within an ultrasound image into the detection of locations with tissue deformation caused by needle penetration. The primary strategy is to compare the changes between a previously recorded baseline frame and the present frame, allowing for the identification of the largest linked region, which serves as an indicator of the needle tip’s position. The centroid of this region is then determined as the exact location of the needle’s tip. By doing frame-by-frame comparisons, the approach successfully filters out unnecessary information while keeping key image features and ensuring high precision in surgical needle localization within the complicated tissue environment.

The ultrasound images are first preprocessed using a variety of procedures such as grayscale conversion, threshold filtering, and morphological opening to remove noise and improve image quality. Once the images have been preprocessed, frame differencing is accomplished by computing the pixel difference between the current frame and a reference frame. This step identifies regions of change, which appear as T-shaped areas in the final difference image. The center of this region is defined as the needle tip’s current position. This position is then further calculated into the physical unit of length using mathematical transformations shown in Equations 1 and 2. These transformations allow the identified location within the ultrasound image to be converted into its corresponding physical coordinates, ensuring accurate spatial alignment of the needle’s position.

$$\text{Output}\left(x,y\right)=\left(P\left(x_{p},y_{p}\right)-G\left(x_{g},y_{g}\right)\right)\times U$$ (1)

$$U=\frac{W_{cm}}{N\left(x_{n},y_{n}\right)-M\left(x_{m},y_{m}\right)}$$ (2)

**FIGURE 2:**
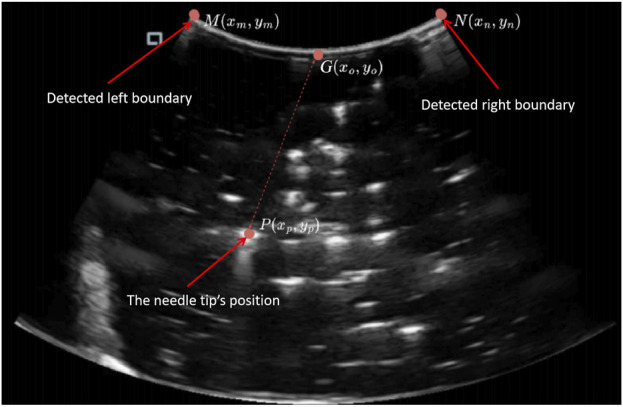
The coordinate transformation calculated in the ultrasound image.

The coordinate transformation is defined by Equation 1, where $P\left(x_{p},y_{p}\right)$ denotes the location of the needle in the ultrasound image as established using the suggested procedure. The physical coordinates in actual space are shown by output (x, y). The origin of the coordinate system, represented by the symbol $G\left(x_{g},y_{g}\right)$ , is located in the middle of the upper boundary of the ultrasonic detection region. The true width of the ultrasonic probe’s detecting region is $W_{cm}$ in Equation 2. The upper edge of the ultrasound detection region’s right and left boundary points are denoted by points N and M . Edge detection techniques are used to precisely determine the reference position and boundary points within the ultrasound image. Establishing this origin is critical for guaranteeing alignment between the image coordinate system and its physical equivalent, allowing for precise spatial mapping as shown in Figure 2. In both Equations 1 and 2, the parameter U represents the real-world distance indicated by each pixel in the ultrasound image, expressed in centimeters.

Occasionally, unexpected problems could occur and affect the needle tip localization accuracy. To overcome this, the ultrasound system has a manual masking function that enables for the selection of a specific region of interest (ROI) within the image. A region of interest (ROI) is a square area measuring 2 cm by 2 cm. This size provides enough space to accurately identify the needle tip while keeping a focused area for precision investigation. The chosen ROI minimizes interference from unnecessary structures and other noise outside the designated area, ensuring that the algorithm functions within a restricted space. This setup improves needle tip localization accuracy while preserving computational efficiency. Normally, the system can accurately identify the needle tip within the ROI. As the needle tip position is corrected after each 1 cm step, the ROI can update itself to keep the needle tip centered. However, in the rare case that the needle tip goes beyond the ROI, the system provides the manually masking function. Manual masking allows operators to choose the area in which the algorithm should run for needle tip recognition. This specified technique eliminates potential biases and inaccuracies caused by unexpected problems in the ultrasound data. Consequently, this feature enhances the system’s overall robustness and reliability during clinical procedures.

Using the above algorithm for needle tip positioning, the system scans the cross-section of the tissue in steps of 1 cm during needle insertion. The scanning moves forward with the needle tip. During insertion, the needle shaft may bend. Currently the ultrasound tracking system focuses primarily on the needle tip’s position. Although bending is a known issue, the system is designed to follow the surgeon’s scanning pattern, prioritizing the accurate tracking of the needle tip within the tissue. The platform currently addresses this by adjusting the control approach to keep the needle tip as the focal point, even when bending occurs.

### 3.2 The robotics control system

**FIGURE 3:**
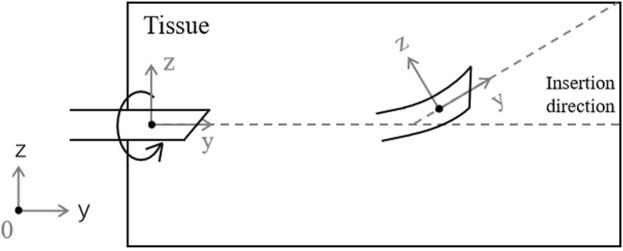
A superimposed bicycle-like nonholonomic model proposed for the bevel tip’s needle.

**FIGURE 4:**
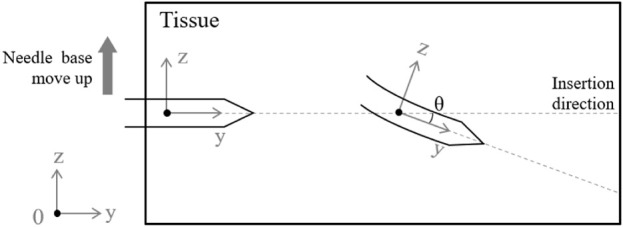
The position of the needle tip is adjusted by manipulating the needle base.

The trajectory of the surgical needle can deviate significantly in complex puncture environments due to a variety of variables, which can cause errors between the expected and actual positions of the needle tip. A bicycle model, with the needle’s tip representing the front wheel and base representing the back, can be used to simulate the behavior of the needle during insertion as shown in Figure 3 (Webster et al., 2006). When the needle is first inserted, it usually follows the pre-planned path, gradually getting deeper along the desired path. Nevertheless, the needle experiences deflection as it penetrates deeper into the tissue due to internal pressure and frictional forces from the surrounding tissue. This deflection causes an error from the planned trajectory. By dynamically altering its curvature, the needle tip can be steered towards the goal, similar to how a unicycle model would be, and the error between its current position and the desired trajectory can be minimized. In our platform, a symmetrical needle that mirrors those used in surgical procedures is used. The position of the needle tip can be controlled by adjusting the needle base position, as shown in Figure 4.

Because of the significant nonlinearity inherent in the steering process, traditional control systems are less capable of handling the complications caused by changing parameters. Fuzzy control offers an effective way to handle variations in system parameters and takes into account the nonlinear character of the system in this situation. Fuzzy control, in contrast to conventional control techniques, achieves precision regulation without the need for a precise and intricate mathematical model. Instead, it employs heuristic principles to manage uncertainties and non-linearities, facilitating resilient performance under varying circumstances. This feature makes fuzzy control particularly helpful for intricate puncture procedures, where it is hard to establish an accurate mathematical model because of the unexpected nature of tissue characteristics and the dynamic interactions that occur during needle insertion.

**FIGURE 5:**
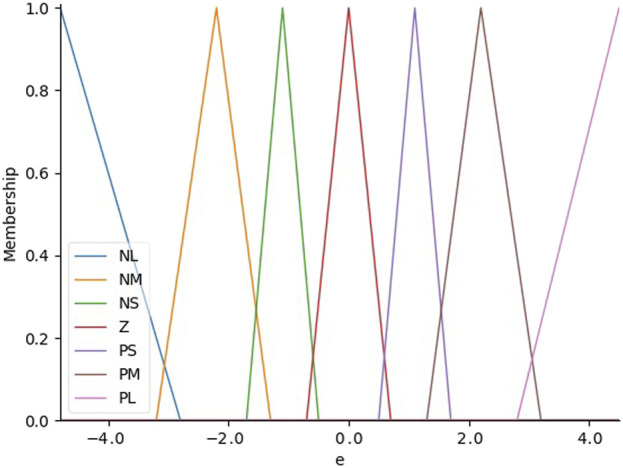
The distribution of input membership functions which are used to represent varying degrees of positional deviation.

Then a special-designed fuzzy control algorithm is employed to determine the appropriate control mode for the robotic system to reduce the error in this system. This algorithm adjusts the “front wheel” of the needle in real-time, allowing for continuous correction of the needle’s path. Consequently, the system ensures that the needle accurately follows the intended trajectory and converges on the desired target point within the tissue, thereby improving overall precision and safety during the procedure. The positional error is the main input variable for the fuzzy controller in the controller architecture, and the corresponding output is the control mode of the puncture manipulator. A triangular membership function is utilized for input fuzzification, which successfully classifies the positional error into seven different linguistic terms: Negative Small (NS), Negative Medium (NM), Negative Large (NL), Positive Medium (PM), Positive Small (PS), Zero (Z), and Positive Large (PL). These membership functions are used to represent varying degrees of positional deviation. Based on modeling results and analysis, a deviation of 0.5 mm is defined as a zero offset, while the deviations between 0.3 mm and 1.7 mm are categorized as small offset, the deviations between 1.3 mm and 3.2 mm are categorized as medium offset and the deviations above 2.8 mm are categorized as large offset as shown in Figure 5. In our proposed system, this input value is the bias between the predefined trajectory and the current real needle tip position.

The fuzzy controller uses the established rule base and the related input linguistic terms to calculate the output linguistic variables after fuzzifying the input values. This process generates control rules that guide the movement of the needle steering. In our scenario, we make adjustments to the position of the needle base in order to control the needle tip’s position within the tissue and compensate for bias. These adjustments are made in both the x and z directions, as the y direction, representing the insertion depth, is consistently set to 1 cm. Therefore, only the x and z directions require adjustment. For example, in the x direction, rules are shown as follows. The control of the z direction is the same to the x direction.

The fuzzy set is transformed into an exact output value for defuzzification using the Center of Gravity approach. Following the fuzzification of the input values, the fuzzy controller uses the rule base and the related input linguistic words to determine the output linguistic variables. First, using the applied fuzzy rules to create the fuzzy set. The centroid of this fuzzy set is then calculated using the membership function as a distribution function. Equation 3 provides a mathematical representation of this process.

$$\text{Output}\left(z\right)=\frac{\int_{\text{all }z}\mu \left(z\right)\cdot z\;dz}{\int_{\text{all }z}\mu \left(z\right)\;dz}$$ (3)

In Equation 3, μ(z) indicates the value of the membership function at a particular z, and Output(z) indicates the final defuzzified value. The formula shows the weighted contribution of each individual value in the set, with the numerator being the integral of the membership function multiplied by its corresponding output value, z. Alternatively, the denominator is the entire mass of the fuzzy set, which is the sum of the values of the membership function. The defuzzified output, or Output(z) , which represents the centroid of the fuzzy set, is produced using the formula above. This value serves as the final control mode applied to the regulation of the needle steering, translating the fuzzy inference into precise control actions for the manipulator which is the distance of the robotics movement to adjust the current position to the predefined trajectory. For example, if the bias of the needle tip position obtained from ultrasound system is +1.5 mm in x direction. It is categorized to both PS and PM for 50% each. After going through the fuzzy rule and defuzzification, the final control output is +1.25 cm which is to move the needle base to plus 1.25 cm in x direction.

### 3.3 The camera visual tracking system

This subsystem is developed based on previous research publication (Li et al., 2024). A brief overview of the camera-based visual tracking system and its integration into the current framework is provided to elucidate its role in the system architecture. The primary objective of the camera visual tracking system is to support the ultrasound-based tracking system by providing complementary data for accurate needle tip localization. Additionally, the system captures the entire bending profile of the needle, extending from its base to the tip. This bending information is crucial for determining the needle’s curvature and evaluating the insertion trajectory. Understanding the bending curve is valuable for ensuring procedural safety, as it allows for the early detection of any abnormal deflections that may pose risks during insertion. The system also offers real-time visual feedback to the clinician, enabling comprehensive monitoring of the needle’s path and the patient’s condition throughout the procedure. This multi-functional role significantly enhances the system’s ability to ensure precision and safety during complex needle steering tasks.

The system consists of three stages: camera calibration, visual tracking within the camera’s coordinate frame, and coordinate transformation from the camera’s frame to the robot’s base frame. Camera calibration is used to ensure accurate tracking of the needle base, which is attached to the robot’s end effector. The technique compensates for any inaccuracies caused by camera lens defects and sensor alignment inconsistencies. To address these distortions, an intrinsic matrix is developed to compensate the mismatched scaling factors between the x and y-axes. The matrix is obtained using the “Kalibr”calibration toolbox after capturing video footage of a checkerboard calibration pattern from multiple angles. This step is fundamental for establishing accurate positional references within the camera’s coordinate system, thereby facilitating reliable tracking throughout the procedure.

Following calibration, visual tracking is carried out using the Channel and Spatial Reliability Tracker (CSRT), an advanced tracking algorithm noted for its robustness and versatility. The CSRT method incorporates channel dependability weights into the classic correlation filter framework, improving its capacity to adapt to changes in the look of the target. During this stage, the camera takes RGB and depth video streams, which are then filtered spatially, temporally, and hole-filling to remove noise and recover missing depth values. This multi-step filtering procedure is critical to keeping stable and smooth visual tracking. Because it ensures that the needle base would be consistently recognized and located.

**FIGURE 6:**
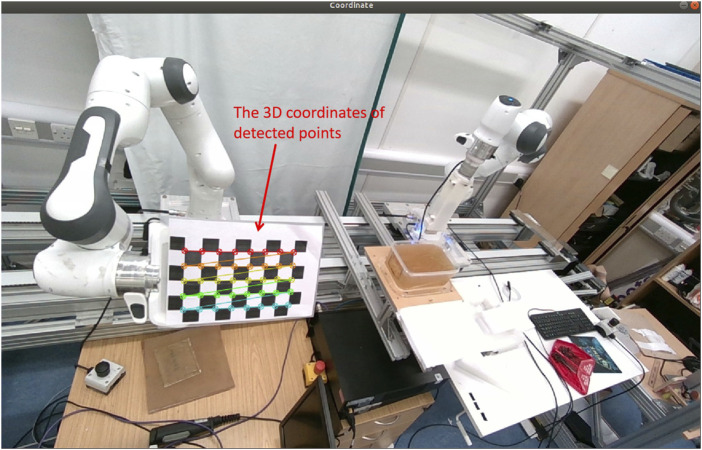
By choosing ten sets of the same point from ten different pose sets of location for robotics end effector and camera, the 3D transformation matrix could be calculated.

The coordinates of the needle base need to be converted from the coordinate system of the camera into the base coordinate system of the robot in order to allow for precise robotic control. As seen in Figure 6, this transformation is achieved by building a transformation matrix using a variety of corner points from a calibration checkerboard that is attached to the end-effector. By eliminating anomalous data points from the matrix, the Random Sample Consensus (RANSAC) technique improves the precision of the coordinate transformation. In order to ensure correct spatial alignment, the calculated transformation matrix makes it possible to precisely transfer the needle’s position from the perspective of the camera into the robotic base frame. This transformation is crucial for the system’s capacity to guide the needle along the desired trajectory, allowing for precise control during the insertion procedure.

## 4 Experiment and result

### 4.1 The experimental setup

**FIGURE 7:**
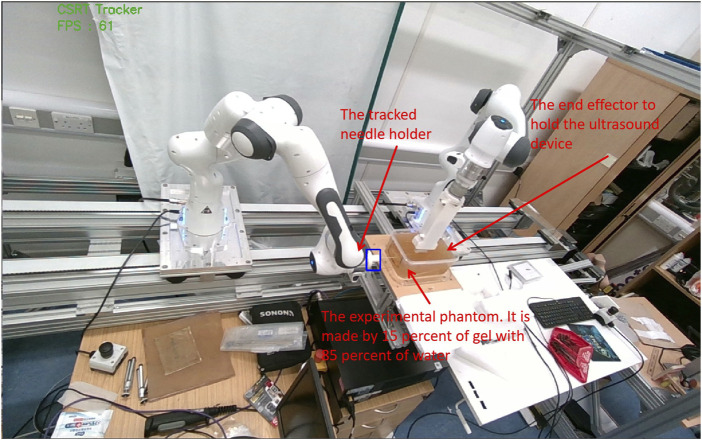
The setup of the experimental platform.

The experimental apparatus is illustrated in Figure 7. The setup comprises two Franka Emika robots, each equipped with a specialized end-effector: one designed to manipulate the needle and the other to operate a mobile ultrasound device. Additionally, the experimental configuration incorporates three laptops. Two of these computers are designated for executing the low-level control system, which receives commands from the primary control system to govern the robotic arms, enabling needle steering and phantom scanning, respectively. The third laptop is allocated for running the three subsystems responsible for guidance and high-level decision-making throughout the needle insertion procedure.

The online experiments were replicated eight times. For each iteration, a target point within the phantom was selected. Subsequently, the robotic system initiated needle insertion from the designated starting point, advancing a brachytherapy needle 8 cm into the phantom. Simultaneously, the visual tracking system monitored the trajectory of the end effector, which secured the needle base throughout the steering process. As the needle tip navigated within the phantom, the ultrasound system located and visualized its position post-insertion into the tissue. Following each insertion step, the current coordinates of the needle tip were extracted from the ultrasound image and utilized to adjust the control parameters for the subsequent step.

### 4.2 The result and evaluation

**FIGURE 8:**
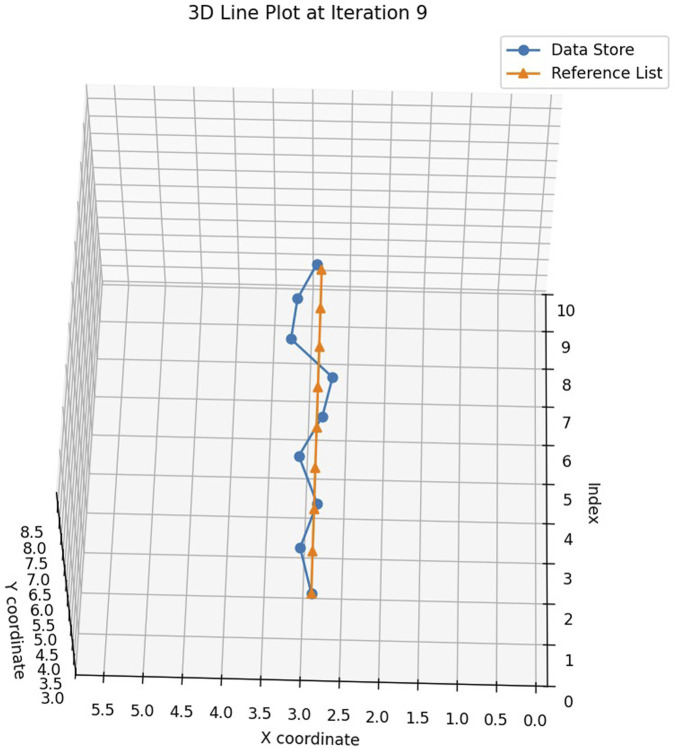
The visualization of the needle tip inside the tissue.

**FIGURE 9:**
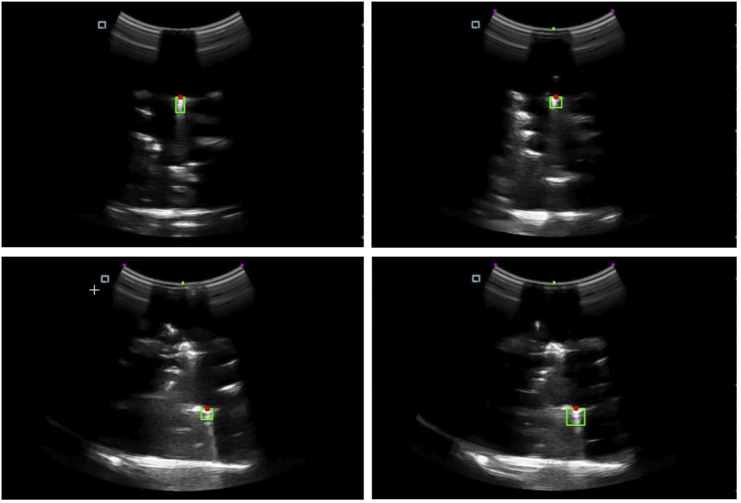
The needle tip localization in the ultrasound image. The green area indicates the region containing the needle tip, while the red dot marks the detected needle tip position.

The study conducted a series of experiments to evaluate the performance of the robotics needle insertion platform. The experiments comprised eight sets of data, each containing real trajectory data points and their corresponding predicted positions. The visualization of a single trial from the experiment is presented in Figure 8. The visual recognition results of ultrasonic device used in the experiment are shown in Figure 9. All measurements were recorded in centimeters. The evaluation metrics utilized in this study include Root Mean Square Error (RMSE), Mean Euclidean Distance, Final Position Error, and Maximum Error as shown in Table 1.

**TABLE 1:** The experimental result and evaluation.

Set	RMSE (cm)	Mean euclidean distance (cm)	Final position error (cm)	Maximum error (cm)
1	0.2690	0.2451	0.2920	0.4123
2	0.3075	0.2830	0.3064	0.4723
3	0.1986	0.1794	0.1471	0.2860
4	0.3641	0.3285	0.3570	0.5894
5	0.2435	0.2184	0.2307	0.3968
6	0.2320	0.2115	0.2309	0.3256
7	0.3235	0.2957	0.3574	0.4836
8	0.3426	0.3107	0.3517	0.5103
**Average value**	**0.2851**	**0.2590**	**0.2842**	**0.4345**

The experimental results show that the proposed system demonstrates promising performance in needle steering process. The mean RMSE of 0.2851 cm indicates that, on average, the predicted trajectories deviate from the actual paths by less than 3 mm. Compared to the system proposed by (Bernardes et al., 2024), our system achieves similar accuracy while utilizing a low-cost imaging device which is to replace the high-cost MRI with a low-cost mobile ultrasound device. This level of accuracy shows that the system could be suitable to be used in the real needle steering scenario. The Mean Euclidean Distance, with an average of 0.2590 cm, corroborates the RMSE findings, providing a complementary perspective on the model’s overall accuracy. The slight difference between these two metrics (RMSE being approximately 10% larger) is consistent with theoretical expectations, as RMSE tends to penalize larger errors more heavily.

An intriguing aspect of the results is the Final Position Error, which averages 0.2842 cm across all sets. This value is close to the mean RMSE, suggesting that the model’s performance at the trajectory endpoints is consistent with its overall accuracy. The Maximum Error metric provides valuable insights into the model’s behavior under challenging conditions. With a mean value of 0.4345 cm, it indicates that even in the worst cases, the accuracy of the needle tip generally remain within half a centimeter of the pre-defined trajectory. However, the fact that this value is significantly higher than the other metrics (approximately 52% larger than the mean RMSE) highlights the presence of occasional larger deviations. This also shows that at this stage, under occasional circumstances, the stability of the system needs to be further improved.

Examining the performance across different experimental sets reveals notable variations. Set 3 exhibits the best performance with the lowest RMSE (0.1986 cm) and Maximum Error (0.2860 cm), indicating particularly accurate control under those specific conditions. Conversely, Set 4 presents the highest RMSE (0.3641 cm) and Maximum Error (0.5894 cm), suggesting the presence of more challenging needle insertion scenarios. This variability indicates the importance of understanding the factors that influence prediction accuracy across different contexts and the potential to further improve the controller to raise the accuracy for needle insertion. The consistency between the Mean Euclidean Distance and the RMSE across all sets (with the Mean Euclidean Distance consistently about 9%–10% lower than the RMSE) suggests a relatively stable error distribution. This stability is a positive attribute of the model, indicating that its performance characteristics are maintained under most of the experimental conditions.

The result shows a high accuracy of needle steering process with the use of the proposed dual-arm robotics system and also provides a solid foundation for future improvement of the system controller. Compared to the system proposed by (Bernardes et al., 2024), our system utilizes a low-cost ultrasound device for visual guidance while achieving a high accuracy of 0.285 cm. The generally low error rates across all metrics demonstrate the system’s potential for practical applications. The experimental results indicate that the system achieves overall high accuracy in most cases, with sub-centimeter average errors. The model’s consistent performance between overall trajectory and endpoint prediction is particularly noteworthy. While the occasional larger deviations reflected in the Maximum Error metric suggest room for improvement, the overall performance of the system is promising for identifying the potential for the system to be applied in real needle steering scenario. However, the variations observed between different experimental sets, particularly in terms of Maximum Error, highlight areas for potential improvement. Future research could focus on identifying the underlying factors contributing to these variations and developing control strategies to enhance the system’s robustness across a wider range of scenarios.

## 5 Conclusion

This paper presents the design and implementation of a dual-arm visual-servo robotics system for assisting needle steering in prostate brachytherapy, offering a innovative solution for enhancing the accuracy and safety of minimally invasive procedures. The system integrates visual tracking using both ultrasound and camera imaging, robotic control utilizing two Franka Emika arms, and a portable ultrasound scanning system into a unified framework. This combination aims to achieve precise steering of brachytherapy needles into the phantom and evaluate the system’s performance in simulated scenarios. The key contributions of this work include the development of a multi-modal visual tracking system, implementation of a fuzzy logic control algorithm for adaptive needle steering, and the design of a coordinated dual-arm robotic system. The integration of camera-based visual tracking provides complementary data and safety monitoring, enhancing the overall reliability of the system.

To the best of our knowledge, this is the first instance where an ultrasound and a camera are integrated with a dual-arm robotic system for needle insertion in a brachytherapy setting. Experimental results demonstrate the system’s capability to achieve high accuracy in both tracking and controlling the needle trajectory. In the future work, we plan to focus on investigating advanced control strategy and further improving the ultrasound image quality and recognition algorithm. Combining the current algorithm and data with modern neural network to develop a new robust control and visual recognition system is our goal for the next stage. It aims to provide a more robust robotics system and push research to the real surgical scene.

## Data Availability

The original contributions presented in the study are included in the article/supplementary material, further inquiries can be directed to the corresponding author.
